# Efficacy of an electronic cognitive behavioural therapy program developed and delivered via the Online Psychotherapy Tool for mental health problems related to the COVID-19 Pandemic

**DOI:** 10.1192/j.eurpsy.2023.1901

**Published:** 2023-07-19

**Authors:** E. Moghimi, M. Omrani, A. Shirazi, J. Jagayat, C. Stephenson, N. Alavi

**Affiliations:** 1 Psychiatry, Queen’s University, Kingston, Canada; 2 Psychiatry, OPTT inc, New York, United States; 3 OPTT inc, Toronto; 4 neuroscience, Queen’s university, Kingston, Canada

## Abstract

**Introduction:**

Lockdowns and social distancing resulting from the COVID-19 pandemic have worsened population mental health and made it more difficult for individuals to receive care. Electronic cognitive behavioural therapy (e-CBT) is a cost-effective and evidence-based treatment that can be accessed remotely. The objective of the study was to investigate the efficacy of online psychotherapy during the pandemic.

**Objectives:**

The purpose of the present study was to develop and administer an e-psychotherapy program for patients with depression and anxiety d), affected by the COVID-19 pandemic . The program aimed to significantly reduce stress and psychological distress in patients, from pre- to post-intervention.

**Methods:**

Participants (n = 59) diagnosed with MDD and/or GAD, whose mental health symptoms initiated or worsened during the COVID-19 pandemic. The online psychotherapy program focused on teaching coping, mindfulness, and problem-solving skills. Symptoms of anxiety and depression, resilience, and quality of life were assessed.

**Results:**

From the participants assessed for eligibility, n = 14 did not meet the inclusion and exclusion criteria and n = 7 declined to participate. As a result, n = 59 participants commenced the study. In total, 21 participants dropped out of the study (n = 11 from Weeks 1-3, n = 7 from Weeks 4-6, and n = 3 at Week 7), and 38 participants completed the study. The large majority of the total sample identified as women (n = 41, 69%). Two participants identified as Other and both dropped out of the treatment at Weeks 4 and 6, respectively. The average age of the sample was 32.26 (SD = 12.67). No significant differences were observed at baseline for any demographic variables or scores of treatment completers and dropouts . A significant difference was observed between the number of sessions completed by those who dropped out and those who finished the program (p < 0.001). On average, treatment dropouts completed approximately 41% of the treatment before dropping out.

Participants demonstrated significant improvements in symptoms of anxiety (p = 0.023) and depression (p = 0.029) after the intervention. Similar trends were observed in intent-to-treat analysis. No significant differences were observed in resilience and quality of life measures.

**Conclusions:**

The evidence strongly suggests that online psychotherapy can supplement the current care model. Although no changes in quality of life or resilience were reported, these findings may be due to the persistent environmental challenges that are outside the normative levels observed pre-pandemic. While the efficacy of e-CBT has been observed across various populations, it is warranted for future studies to investigate the role of gender in treatment availability and help-seeking.

**Disclosure of Interest:**

E. Moghimi: None Declared, M. Omrani Shareolder of: OPTT inc, A. Shirazi: None Declared, J. Jagayat: None Declared, C. Stephenson: None Declared, N. Alavi Shareolder of: OPTT inc

